# Super-resolution Microscopy – Applications in Plant Cell Research

**DOI:** 10.3389/fpls.2017.00531

**Published:** 2017-04-13

**Authors:** Veit Schubert

**Affiliations:** Leibniz Institute of Plant Genetics and Crop Plant Research, (IPK) GaterslebenSeeland, Germany

**Keywords:** chromosome, nucleus, PALM, plant cell, STED, SIM, STORM, super-resolution

## Abstract

Most of the present knowledge about cell organization and function is based on molecular and genetic methods as well as cytological investigations. While electron microscopy allows identifying cell substructures until a resolution of ∼1 nm, the resolution of fluorescence microscopy is restricted to ∼200 nm due to the diffraction limit of light. However, the advantage of this technique is the possibility to identify and co-localize specifically labeled structures and molecules. The recently developed super-resolution microscopy techniques, such as Structured Illumination Microscopy, Photoactivated Localization Microscopy, Stochastic Optical Reconstruction Microscopy, and Stimulated Emission Depletion microscopy allow analyzing structures and molecules beyond the diffraction limit of light. Recently, there is an increasing application of these techniques in cell biology. This review evaluates and summarizes especially the data achieved until now in analyzing the organization and function of plant cells, chromosomes and interphase nuclei using super-resolution techniques.

## Introduction

Light microscopy of DNA and proteins specifically fluorescently labelled by FISH and immunostaining, respectively, as well as live cell imaging based on fluorescent recombinant proteins significantly increased our knowledge concerning cell organization and function, and is an important advantage compared to electron microscopy.

However, due to the diffraction limit of light as defined by Abbe (1873) the spatial resolution of light microscopy including conventional fluorescence techniques is restricted, and reaches only ∼200 nm laterally and ∼600 nm in the axial dimension in biological specimens (Pawley, 1995). Thus, this limited resolution did not allow identifying single molecules and structures with the resolution achieved by electron microscopy.

Recently, to overcome this restriction and to bridge the resolution gap between light and electron microscopy the so-called super-resolution (also referred as optical nanoscopy) techniques SIM, PALM, STORM, and STED offering new insights into molecular structures, interactions and functions were developed. These “subdiffraction” methods can be divided into two different principles: (i) localization of individual fluorophores in the specimen with subdiffraction precision (PALM, STORM), and (ii) structuring the illumination light to collect high spatial frequencies in the image that contain high resolution information (SIM, STED) (Rego et al., 2012).

To honor the establishment of these stunning methods as PALM/STORM and STED the Nobel Prize in Chemistry 2014 was awarded to Eric Betzig, William Moerner and Stefan Hell, respectively (Gevaux, 2014; Möckl et al., 2014; Stelzer, 2014). The meanwhile widely applied SIM technique has mainly been developed by the late Mats Gustafsson (Gustafsson, 2005; Gustafsson et al., 2008).

In addition to special super-resolution microscope systems established in different research groups, since 2004 also commercial systems are produced by companies as Leica Microsystems (Leica TCS SP8 STED 3X - STED; Leica SR GSD 3D microscope - STORM), Carl Zeiss (Elyra S.1 - SIM; Elyra P.1 - PALM; Elyra PS.1 - combination of SIM and PALM), Nikon (N-SIM; N-STORM; combination of N-SIM and N-STORM) and GE Healthcare Life Sciences (DeltaVision OMX System - SIM; DeltaVision Localization Microscopy System - STORM; combination of SIM and STORM).

Excellent reviews describing and comparing the different super-resolution light microscopy methods (Schermelleh et al., 2010; Agrawal et al., 2013; Allen et al., 2014; Komis et al., 2015b; Nienhaus and Nienhaus, 2016) already exist. These techniques were applied successfully in cell biology (Rouquette et al., 2010; Han et al., 2013; Fornasiero and Opazo, 2015) at fixed and live specimens from both pro- and eukaryotes and helped to discover new structures.

Compared to animal tissues and due to varying refractive indices of plant cell organelles inducing spherical aberrations and light scattering, plant cell imaging is particularly challenging (Komis et al., 2015b). Nevertheless, the applications in this field are also increasing. Here I summarize and evaluate the recent achievements in plant cell research applying different super-resolution techniques.

## Applications in Plant Cell Research

Similar as in other organisms analyzing cell structures of plants is essential to understand biological functions. Thus, increasing efforts are undertaken to apply super-resolution techniques in plant cell research.

Investigations were performed on structures outside and inside of the nucleus, and on condensed chromosomes during cell division (**Table 1**). Most results were obtained from fixed material, but also live cell imaging based on fluorescently tagged proteins was performed.

**Table 1:** Applications of super-resolution methods to analyze plant cell structures.

Structures/cell stages analyzed	Results	Species^a^	Methods	Reference
*Cellular components outside of the nucleus/chromosomes*				
Pollen	Analysis of pollen surface texture and shape	2	SIM	Sivaguru et al., 2012
Cellulose microfibrils	Analysis of the cellulose microfibril architecture in the cell walls of onion-bulb scale epidermal cells	3	STORM	Liesche et al., 2013
Cortical microtubules	Organization and development of cortical microtubules in living hypocotyl epidermal cells	4	SIM	Komis et al., 2014
Cortical microtubules	Vizualization of cortical microtubules in living cotyledon, petiole and root cells	4	SIM	Komis et al., 2015a
Cortical microtubules	Organization and quantitation of cortical microtubules in different root cells	4	STORM	Dong et al., 2015
Endosomes	Imaging and quantification of early and late endosomes during root hair formation	4	SIM	von Wangenheim et al., 2016
Plasmodesmata	Organization of plasmodesmata in leaf parenchyma cells	11	SIM	Fitzgibbon et al., 2010
Plasmodesmata	Arrangement of plasmodesmata and callose in leaf cells	4	SIM	Bell and Oparka, 2011
Plasma membrane	Tracking of individual membrane protein motions in living root epidermal cells	4	PALM	Hosy et al., 2015
Plasma membrane	Detection of polar-competent PIN protein clusters responsible for auxin transport in the apical plasma membrane of living root cells	4	STED	Kleine-Vehn et al., 2011
Perinuclear cell region	Localization of perinuclear actin in living tobacco cells (BY-2)	11	PALM	Durst et al., 2014
Plasmodesmata and virus proteins	Identification of callose and viral movement proteins in the central cavities of complex plasmodesmata in leaf epidermal cells	4	SIM	Fitzgibbon et al., 2013
Plasmodesmata and virus proteins	Localization of plant virus movement proteins in plasmodesmata	10	SIM	Tilsner et al., 2013
Sieve element reticulum and virus proteins	Arrangement of the sieve element reticulum, callose, and cellulose, and identification of potato virus X filaments in phloem cells	10, 11	SIM	Bell et al., 2013
Membrane structures and virus filaments	Visualization of membrane structures of pseudoviral replication complexes and individual potato virus X filaments in leaf cells	10	SIM	Linnik et al., 2013
Plasmodesmata and virus proteins	Localization of RTNLB proteins in the the central desmotubule of plasmodesmata and their colocalization with tobacco mosaic virus movement proteins	4	SIM	Knox et al., 2015
Membrane domains and fungus structures	Identification of extrahaustorial membrane domains and proteins in living leaf cells after *Phytophtora infestans* infection	10	SIM	Bozkurt et al., 2014
*Chromatin and protein organization in interphase nuclei*				
Somatic interphase	Chromatin ring formation of satellite DNA	14	SIM	Ribeiro et al., 2017
Interphase nuclei, mitosis	Distribution of histone H3K4me2, H3K9me2 and H3K27me3 in holocentric chromosomes	9	SIM	Heckmann et al., 2013
Interphase nuclei	Detection of active and inactive RNAPII in the proximity of B chromosome chromatin	17	SIM	Ma et al., 2017
Meristematic nuclei	Determination of the spatio-temporal distribution of rDNA during replication	4	SIM	Dvořáčková et al., in press
Meristematic and endopolyploid nuclei	Distribution and quantification of active and inactive RNAPII within euchromatin	4, 17	SIM	Schubert, 2014
Endopolyploid nuclei	Distribution and quantification of active and inactive RNAPII within euchromatin	4	SIM, PALM	Schubert and Weisshart, 2015
Interphase nuclei	Co-localization of the transcript elongation factor SPT5 and RNAPII within euchromatin	4	SIM	Dürr et al., 2014
Interphase nuclei	Co-localization between the transcript elongation factors SPT6L, ELF7 and RNAPII	4	SIM	Antosz et al., 2017
Differentiated nuclei	Distribution of SMC3 and CAP-D3; eu- and heterochromatin characterization in endopolyploid nuclei	4	SIM	Schubert et al., 2013
*Chromatin/protein organization along condensed chromosomes*				
Pachytene	Mitochondrial and plastidal DNA localization in B chromosomes	17	SIM	Klemme, 2013
Somatic metaphase	Accumulation of chloroplast- and mitochondria-derived sequences in B chromosomes	1	SIM	Ruban et al., 2014
Somatic metaphase	Localization of satellite repeats along holocentric chromosomes	13, 14, 15, 16	SIM	Ribeiro et al., 2017
Pachytene	Evaluation of the cytosine methylation status of satellite DNAs	5	SIM	Zakrzewski et al., 2014
Somatic metaphase	Detection of sister chromatid exchanges	7	SIM	Vu et al., 2014
Somatic metaphase	Sister chromatid exchange arrangement in mono- and holocentric chromosomes	9, 17	SIM	Schubert et al., 2016b
Somatic metaphase	Localization of H2AThr120ph and H3S10ph at chromosome arms	6	SIM	Sousa et al., 2016
Meiosis	Analysis of the synaptonemal complex formation and the progression of meiotic chromosome synapsis	7	SIM	Phillips et al., 2012
Meiosis	Analysis of the synaptonemal complex organization and interlock formation	19	SIM	Wang et al., 2009
Zygotene	Colocalization of AFD1 and ASY1 during the synaptonemal complex formation	19	SIM	Gustafsson et al., 2008
Mitosis	Visualization of 3xHMG-box proteins at somatic chromosomes	4	SIM	Antosch et al., 2015
*Centromeres*				
Somatic metaphase	Deviating centromere chromatin organization in A and B chromosomes	17	SIM	Banaei-Moghaddam et al., 2012
Metaphase I	Detection of CENH3 at the centromeres of bi- and univalents	17	SIM	Cuacos, 2013
Interphase nuclei, mitosis, meiosis	Chromatin ring formation at centromeres	1, 4, 7, 17, 18	SIM	Schubert et al., 2016a
Interphase nuclei, mitosis	Co-localization of CENH3 and centromere-specific repeats in holocentromeres	15	SIM	Marques et al., 2015
Interphase nuclei, mitosis, meiosis	Co-localization of tubulin, CENH3, CENP-C and centromere-specific repeats in holocentromeres	15	SIM	Marques et al., 2016
Somatic inter- and metaphase	Localization of CENH3 and centromeric repeats along holocentric chromosomes	13, 14, 15, 16	SIM	Ribeiro et al., 2017
Interphase nuclei, mitosis, meiosis	CENH3 amount measurements based on fluorescence intensities	17	SIM	Schubert et al., 2014
Somatic metaphase	Localization of CENH3 along holocentric chromosomes	15	SIM	Cabral et al., 2014
Mitosis, meiosis	Co-localization of the two CENH3 variants	7	SIM	Karimi-Ashtiyani et al., 2015
Somatic inter- and metaphase	Intermingled co-localizaton of αCENH3 and βCENH3	7	SIM	Ishii et al., 2015
Interphase nuclei	Intermingled colocalization of *A. thaliana* and *Zea mays* CENH3s	4	SIM	Maheshwari et al., 2016
Somatic metaphase	Intermingled co-localizaton of αCENH3 and H2AThr120ph	7	SIM	Demidov et al., 2014
Somatic metaphase	Localization of CENH3 and H2AThr120ph in holokinetic chromosomes	9	SIM	Jankowska et al., 2015
Mitosis	Co-localization of α and βCENH3, H2AThr120ph and tubulin at holo- and monocentromeres	9, 7	SIM	Wanner et al., 2015
Meiosis	Co-localization of CENH3, H2AThr120ph and tubulin at holocentromeres	9	SIM	Heckmann et al., 2014
Somatic metaphase	Co-localization of CENH3 and α-kleisin in mono- and holocentromeres	9, 7	SIM	Ma et al., 2016
Meristematic nuclei	Co-localization of CENH3 and KNL2	4	SIM	Lermontova et al., 2013
Root tip nuclei	Co-localization of CENH3 and KNL2	4	SIM	Sandmann et al., 2017
Interphase nuclei, mitosis	Co-localization of CENH3 and GIPs at centromeres	4	SIM	Batzenschlager et al., 2015
Somatic metaphase	Co-localization of both CENH3 variants, H2AThr120ph and H3S28ph in polycentric chromosomes	8, 12	SIM	Neumann et al., 2016
Somatic metaphase	Localization of H2AThr120ph and H3S10ph at centromeres	6	SIM	Sousa et al., 2016
*(Sub)telomeres*				
Somatic metaphase	Localization of telomeric repeats in holokinetic chromosomes	9	SIM	Jankowska et al., 2015
Interphase nuclei, mitosis, meiosis	Chromatin ring formation at subtelomeres	1, 4, 7, 17, 18	SIM	Schubert et al., 2016a


*^a^(1) *Aegilops speltoides* (Boiss.) Chennav.; (2) *Agropyron repens* (L.) *P. Beauv.*; (3) *Allium cepa* L.; (4) *Arabidopsis thaliana* (L.) Heynh.; (5) *Beta vulgaris* L.; (6) *Coccinia grandis* (L.) Voigt; (7) *Hordeum vulgare* L.; (8) *Lathyrus sativus* L.; (9) *Luzula elegans* Lowe; (10) *Nicotiana benthamiana* Domin; (11) *Nicotiana tabacum L.*; (12) *Pisum sativum* L.; (13) *Rhynchospora ciliata* (G. Mey.) Kük.; (14) *Rhynchospora globosa* (Kunth) Roem. & Schult.; (15) *Rhynchospora pubera* (Vahl) Boeckeler; (16) *Rhynchospora tenuis Link*; (17) *Secale cereale* L.; (18) *Triticum aestivum* L.; (19) *Zea mays* L.*

Fixed specimens were stained with specific antibodies and/or hybridized with labeled DNA probes. Such experiments were the basis to quantify and colocalize polysaccharides, proteins and DNA.

Live cell imaging has already successfully applied to follow the development of cytoskeleton components, membrane proteins and fungal infection structures.

Meanwhile, in addition to model plants as *Arabidopsis* and tobacco, also cereals and holocentric wild species were investigated by super-resolution microscopy.

Till now, among the different super-resolution techniques most results were achieved by SIM, whereas fewer applications are based on PALM/STORM and STED (**Table 1**).

### Cellular Components Outside of the Nucleus/Chromosomes

Super-resolved imaging was applied to investigate interactions between different cells, cellular organelles and nuclei.

In this field Oparka and co-workers studied especially by using SIM cell-cell interactions by plasmodesmata imaging (Bell and Oparka, 2011, 2015) and localized plant virus proteins therein. Additionally, by other groups cytoskeleton components and membrane structures were analyzed on fixed specimens (**Table 1**). STORM was applied to elucidate the cellulose microfibril organization in onion cells (Liesche et al., 2013) and the cortical microtubule arrangement in *Arabidopsis* roots (Dong et al., 2015).

Live cell imaging using fluorescent tags such as GFP and mCherry fused to genetically encoded marker proteins together with SIM has been applied to follow the development of microtubules in *Arabidopsis* hypocotyl epidermal cells (Komis et al., 2014), but also in cotyledon, petiole and root cells (Komis et al., 2015a). Bozkurt et al. (2014) identified by SIM fungus structures after *Phytophtora* infection in living tobacco leaves. This indicates that SIM is also a very versatile method with a broad application potential in plant live cell research.

Photoactivated Localization Microscopy on living cells was performed to track *Arabidopsis* root membrane proteins (Hosy et al., 2015) and to localize perinuclear actin in tobacco (Durst et al., 2014).

To date only one STED application in plant cell research has been published. Kleine-Vehn et al. (2011) detected polar-competent YFP-labeled PIN protein clusters responsible for auxin transport in the apical plasma membrane of living *A. thaliana* root cells. The high laser power required for STED causing fast bleaching impedes the acquiring of image stacks and longer live cell imaging. In addition, the number of applicable florescence dyes is restricted. The high degree of autofluorescence and the presence of color pigments (e.g., chlorophyll) make plant tissues especially challenging for STED. Obviously, this causes its so far restricted application in plant cell research.

### Chromatin and Protein Organization in Interphase Nuclei

To understand such basic cellular functions as transcription, replication, and DNA repair the organization of chromatin, DNA–DNA, DNA–protein and protein-protein interactions have to be investigated in interphase nuclei. For this aim, super-resolution imaging was performed on nuclei in tissue squash preparations. However, especially the imaging of isolated and flow-sorted nuclei delivered excellent resolutions due to the absence of cytoplasm (**Table 1**).

**FIGURE 1:**
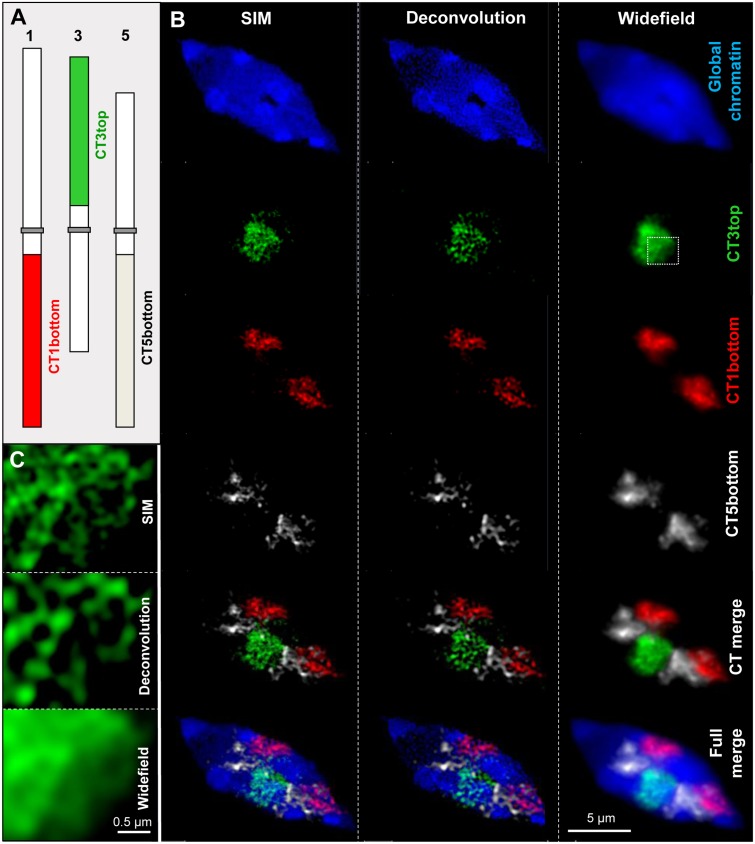
**Chromatin organization in a differentiated 8C *A. thaliana* leaf nucleus.** Three chromosome arm territories (CTs) of chromosomes 1, 3, 5 showing similar size were stained in different colors **(A)**. After FISH and SIM the distribution of the euchromatic arm regions becomes visible beyond the diffraction limit of light, in comparison to the resolution achieved by deconvolution and widefield microscopy **(B)**. The enlarged region of CT3top (rectangle) shows clearly the increased resolution compared to widefield and deconvolution imaging **(C)**.

FISH with differently labeled DNA probes allowed investigating the subchromosomal arrangement of chromatin within cell nuclei (Schubert et al., 2013) (**Figure 1**). Compared to widefield microscopy and deconvolution imaging the increased content of information due to the higher resolution obtained by SIM becomes obvious.

**FIGURE 2:**
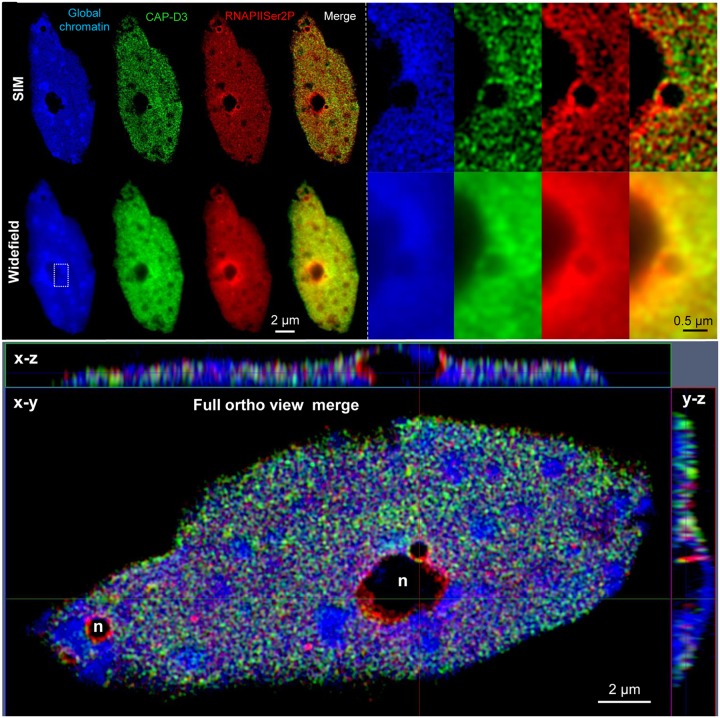
**Distribution and co-localization of active RNAPII (phosphorylated at serine 2) and the condensin subunit CAP-D3 in a flow-sorted differentiated 32C *A. thaliana* leaf nucleus.** After immunostaining with specific antibodies and SIM it becomes visible that both proteins are present within euchromatin, but absent from heterochromatin (dense blue staining) and nucleoli (n) (upper left). Especially the enlarged region (rectangle) shows the increased resolution obtained by SIM compared to widefield microscopy (upper right). The ortho view (below) generated from fully merged SIM image stacks visualizes the nucleus as front (*x–y*) and side (*x–z, y–z*) views. Relative voxel intensity measurements based on the SIM image stack using the Imaris 8.0 (Bitplane) software showed that this nucleus contains ∼19% less RNAPII than CAP-D3 molecules, and that ∼81% of these molecules colocalize.

Several publications show the distribution and colocalization of RNA polymerase II (RNAPII) enzymes and interacting factors in *Arabidopsis* nuclei after labeling with specific antibodies. In addition, the relative and absolute quantification of these molecules in 3D image stacks was performed by SIM and PALM, respectively (Schubert, 2014; Schubert and Weisshart, 2015). **Figure 2** demonstrates the colocalization of RNAPII and the structural condensin subunit CAP-D3 within the euchromatin of an isolated 32C *Arabidopsis* nucleus, and the improved resolution achieved by SIM compared to widefield microscopy.

**FIGURE 3:**
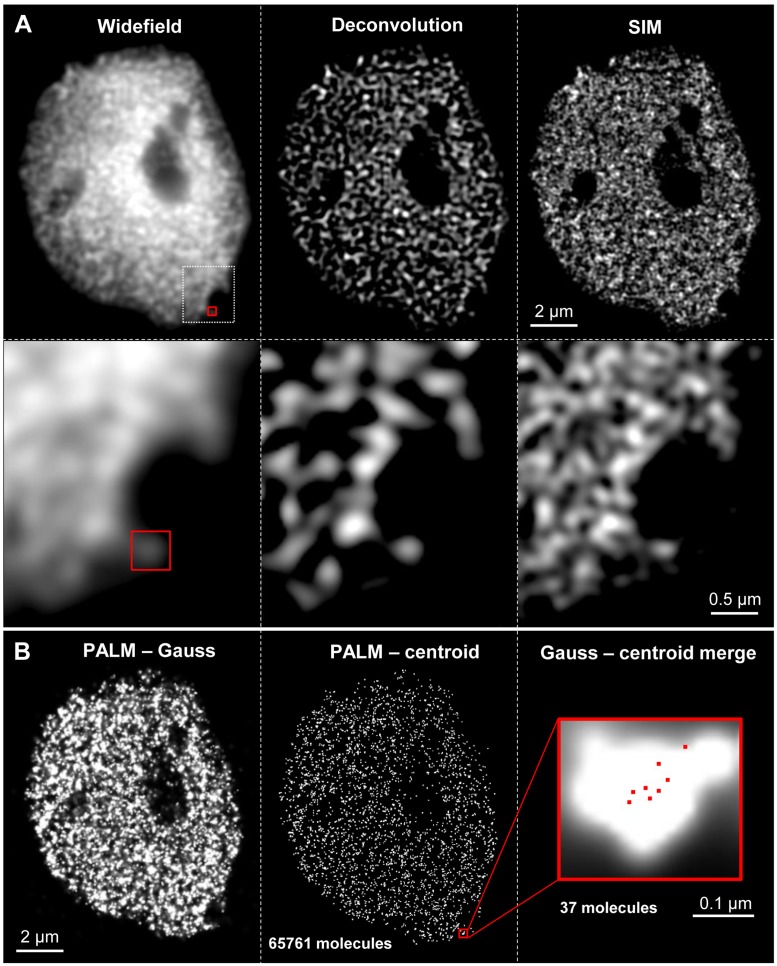
**Distribution and quantification of active RNAPII (phosphorylated at serine 2) molecules in a differentiated 16C *A. thaliana* leaf nucleus.** After immunostaining with specific antibodies SIM **(A)** and PALM **(B)** were performed consecutively. **(A)** Compared to widefield and deconvolution microscopy SIM clearly delivers an increased resolution especially visible in the enlarged region (white rectangle) below. This indicates that RNAPII is distributed network-like within the nucleus. **(B)** After 3D-PALM the resolution becomes further increased when visualized as Gauss distribution (left). Centroid visualization allows the exact localization and counting of single molecules (center). The nucleus with a *z* extension of 1.2 μm contains in total 65761 molecules which were counted in 30 slices of 40 nm. The merged Gauss-centroid view (right) represents the localization of eight single molecules in a single slice of a RNAPII cluster (red rectangle) containing in total 37 molecules in five slices with a total *z* range of 0.2 μm.

The combination of SIM and PALM feasible with systems such as the Elyra PS.1 from Zeiss enables acquiring image stacks by both techniques subsequently. Then, the combination of these stacks allows counting and localizing single molecules within the structures identified by SIM (Schubert and Weisshart, 2015; Weisshart et al., 2016) (**Figure 3**).

### Chromatin and Protein Organization Along Condensed Chromosomes

All organisms perform cell divisions, at which for proper segregation during mitosis and meiosis chromosomes have to be condensed. To better understand this process super-resolution microscopy has been used to analyze the distribution and organization of DNA in cereal supernumerary B chromosomes (Ruban et al., 2014), and of satellite DNA along holocentric chromosomes (Ribeiro et al., 2017) (**Table 1**).

Additionally, the distribution of specific DNA (Zakrzewski et al., 2014) and histone modifications (Sousa et al., 2016) was investigated along condensed chromosomes.

The arrangement of sister chromatids in holocentric chromosomes could be clarified by differential labeling via base analog incorporation during replication followed by SIM (Schubert et al., 2016b).

SIM was also helpful to analyze the synaptonemal complex formation during homologous chromosome paring in prophase I of maize (Gustafsson et al., 2008; Wang et al., 2009) and barley (Phillips et al., 2012).

### Centromeres and (Sub)Telomeres

Centromeres/kinetochores as spindle fiber attachment sites are required for proper chromosome segregation during cell division. Telomeres protect chromosome termini from degradation and fusion. Thus, both are essential to maintain genome stability of organisms.

SIM investigations were helpful to get new insight into the centromere organization during the cell cycle via specifically labeling and co-localizing centromere components such as centromere-specific DNA repeats, kinetochore proteins and histone modifications at centromeric chromatin (**Table 1**).

Especially the finding that phosphorylated histone H2A positive chromatin and different CENH3 variant containing chromatin clusters intermingle to form centromeres has been attained (Demidov et al., 2014; Ishii et al., 2015; Karimi-Ashtiyani et al., 2015), an observation not achievable by refraction-limited widefield resolution. Similar comparative investigations in mono-, poly-, and holocentric plants species provided also new insights into the evolution of centromeres (Wanner et al., 2015; Neumann et al., 2016).

Regarding telomere investigations until now only two publications appeared. One was published about the localization of telomeric repeats in holokinetic *Luzula* chromosomes (Jankowska et al., 2015), a second about chromatin ring formation at subtelomeres in barley (Schubert et al., 2016a).

## Specimen Preparation and the Super-Resolution Methods

All super-resolution techniques are based on imaging fluorescent molecules. Consequently, they are used to label structures and molecules of interest. After fixation of a specimen, which should alter the native structures as less as possible, specific fluorescent affinity probes of preferably small size (FAB fragments of antibodies, nanobodies, snap-tags) may be used for labeling (Fornasiero and Opazo, 2015).

The advantage of genetically encoded and expressed fluorescent proteins (Hedde and Nienhaus, 2014) is that they allow investigating dynamic processes in living cells without fixation artifacts. However, due to phototoxicity live cell nanoscopy is much more challenging than imaging fixed specimens (Fornasiero and Opazo, 2015). Hence, fewer live cell imaging results have been published so far in plant cell research (**Table 1**).

Both structured illumination and localization microscopy may be used for fixed material but also for imaging living cells. Depending on the different super-resolution techniques and the imaging tasks to be performed (e.g., quantification and colocalization of molecules) the specimen preparations have to be adapted accordingly. Staudt et al. (2007) developed a new embedding medium especially useful for STED microscopy to avoid spherical aberrations induced by the refractive index mismatch between the immersion system and the embedding medium of the sample.

### Imaging of Fixed Specimens

The major advantage of SIM is that most preparation and labeling protocols and fluorophores used for widefield fluorescence microscopy are applicable without modification, thus allowing high-throughput experiments. Despite a reliable tissue fixation, the use of high-quality glass slides and especially coverslips (e.g., Marienfeld high precision cover glasses) are important to reach the possible resolution of up to ∼120 nm by a 488 nm excitation. It is quite important to notice that during SIM raw data acquisition an overexposure must be avoided perfectly. Otherwise, artificial not existing structures and shapes can be generated during the SIM calculation.

Using a Zeiss ELYRA PS.1 microscope system the acquiring of image stacks of up to 30 slices at a distance of ∼100 nm at full resolution (∼1024 × 1024 pixel, 80 μm × 80 μm FOV, 100 ms exposure time), which takes ∼4–5 min in a sequential 3-color experiment (see **Figure 1**) are the basis to calculate 3D-SIM image stacks within ∼10-20 min (Weisshart et al., 2016). These stacks may be used for 3D-movie rendering by, e.g., the commercial ZEN (Zeiss) (e.g., Schubert et al., 2013, 2016a) or Imaris (Bitplane) (Neumann et al., 2016; Schubert et al., 2016b) softwares.

To reveal the spatial ultrastructure of cellular components SIM delivers best results after applying FISH and/or immunolabelling at relatively flat (up to ∼10 μm) tissue squashes and spreadings. But especially useful are isolated and flow-sorted cell nuclei free of disturbing cytoplasm (Dürr et al., 2014; Weisshart et al., 2016; Antosz et al., 2017) (**Figure 2**).

SIM image stacks are also useful to determine relative molecule amounts via pixel intensity measurements in organelles like nuclei, and to measure the degree of colocalization of differently labeled molecules (Dürr et al., 2014; Schubert, 2014; Antosz et al., 2017) (**Figure 2**).

Bell and Oparka (2015) developed preparative methods especially for imaging plasmodesmata by SIM on fixed plant tissues.

Practically localization microscopy (PALM, STORM) reaches a circa five-fold higher resolution than SIM. Thus, single molecules may be identified, counted and colocalized in single- and even two-color experiments using, e.g., the fluorescence dyes Alexa488 and Cy5 (Schubert and Weisshart, 2015; Weisshart et al., 2016). However, compared to SIM specimen preparation is more challenging and raw data acquisition and calculations are more time-consuming. To achieve reliable results a high labeling density and efficient photoactivation are required (Fernández-Suárez and Ting, 2008).

2D-PALM may be performed under HILO (Tokunaga et al., 2008), Epi and TIRF illumination (Hedde and Nienhaus, 2014), whereby TIRF will give the best signal, followed by HILO and last Epi. 3D-PALM features dependent on the 3D-technology used to capture ranges between 1.2 and 2.0 μm. This range might be extended by using classical z-scans. For a better signal HILO instead of Epi illumination is recommended (van de Linde et al., 2011). TIRF and for best performance also HILO illumination need the positioning of the specimen very close to the coverslip surface. Thus, the preparation of specimens directly onto coverslips has to be performed. These coverslips may be fixed onto slides by rubber cement and then be handled as usual during the staining and washing procedures. After placing them into coverslip chambers, adding a redox reagent and adjusting the pH to the needs of the dye to achieve efficient photoactivation, PALM can be performed (Weisshart et al., 2016). The redox reagent, e.g., 1% 2-mercaptoethanol in 1x PBS can be used if the fluorescence dyes in the specimens are easily accessible. Otherwise, adding of glucose is required (Olivier et al., 2013; Schäfer et al., 2013).

PALM using fluorescent proteins can be performed in buffers like PBS or Hepes. Acquiring raw data sets takes ∼15 min, followed by a calculation procedure of also ∼15 min if, e.g., isolated nuclei and the Elyra PS.1 is used.

Non-commercial super-resolution microscope setups (Hamel et al., 2014) and commercial systems as the Elyra PS.1 (Weisshart et al., 2016) allow producing SIM and PALM/STORM data subsequently. Thereby, the observed SIM structures can be combined with precise PALM/STORM single molecule localization and counting. This clearly increases the information obtained from the specimens under investigation (**Figure 3**).

### Life Cell Imaging

Expressed fluorescent reporter proteins allow visualizing proteins and structures inside living organisms (Hedde and Nienhaus, 2014). Because the implementation of super-resolution microscopy in live cell microscopy is very challenging and both structured illumination and localization microscopy have different advantages and disadvantages (Schermelleh et al., 2010), to date only few publications describing the dynamics of plant cell components appeared (see above).

Propagating seedlings in coverslip chambers under sterile conditions allow live cell imaging by SIM on roots growing closely along the coverslip. However, due to the fast root elongation via cell extension the imaging time (e.g., in *Arabidopsis*) is limited, because the roots quickly leave (within ∼30 min) the field of view.

The application of hypocotyls circumvents this problem. Samaj and co-workers produced excellent super-resolved movies via SIM by analyzing the microtubule development in *Arabidopsis* hypocotyl epidermal cells by adapting the settings accordingly to acquire the raw data. For tissue etiolation the seedlings were grown in darkness which induces the thinning of the outer epidermal wall and reduces the thickness of the cuticular surface. Then, after mounting the seedlings into aqueous growth medium, effects causing refractive index mismatches can be reduced (Komis et al., 2014, 2015a).

PALM in living cells was performed on *Nicotiana* BY-2 cell cultures after transferring the cells into coverslip chambers (Chamber Slides^TM^, Thermo Scientific) to localize perinuclear actin (Durst et al., 2014).

Single particle tracking by PALM was used for the first time on living plants by Hosy et al. (2015). They mounted *Arabidopsis* seedlings between two coverslips to track plasma membrane proteins.

## Conclusion and Perspective

Currently, most results obtained by super-resolution microscopy in plant cell research are concentrated in the fields of research groups with access to super-resolution microscopes. But it is expected that the applications will extent in future significantly due to the general applicability of super-resolution to analyze biological specimens, so that super-resolution microscopy will become a standard technique also in plant cell research.

This development will be further accelerated by improving and combining the existing super-resolution methods. Especially SIM has the potential for extended applications in the field of live cell imaging. Additionally, SIM methods are under development to excel its to date achieved two-fold increased resolution.

To image also thick fluorescent samples (*Calliphora* salivary glands) SIM was combined with line-scanning to remove disturbing out-of-focus fluorescence background deteriorating the illumination pattern (Mandula et al., 2012). Rego et al. (2012) developed nonlinear SIM, and thus were able to visualize with a ∼ 40 nm resolution purified microtubules, mammalian nuclear pores and the actin cytoskeleton by applying the fluorescent photoswitchable protein Dronpa.

SIM can be applied for live cell imaging in multiple colors by using conventional fluorescent dyes as fast as 11 frames/s (Kner et al., 2009) at intensities of only 1 to 100 W/cm^2^ preventing phototoxicity (Li et al., 2015). Betzig and co-workers extended the resolution of live cell SIM by using an ultrahigh numerical aperture TIRF-SIM and achieved up to 84 nm, and by patterned nonlinear SIM they obtained up to 45-62 nm. By this approach the dynamics of plasma membranes components, mitochondria, actin and the Golgi apparatus in cultured mammalian cells has been imaged (Li et al., 2015). In addition, patterned nonlinear SIM and lattice light sheet microscopy (Chen et al., 2014) were combined to perform 3D live cell imaging beyond the diffraction limit (Li et al., 2015).

Gao et al. (2012) performed *in vivo* karyotyping of somatic chromosomes and identified the dynamics of the cytoskeleton of fibroblasts by combining an ultrathin planar illumination (produced by scanned Bessel beams) with SIM at thick animal specimens. Similarly, based on this technique the dynamics of mitochondria, filopodia, membrane ruffles, intracellular vesicles, and mitotic chromosomes in living cultured cells were investigated (Planchon et al., 2011).

It is expected that also the use of localization microscopy for absolute molecule quantification will be intensified in future, and that the combination with SIM will be increased to employ the advantages of these different nanoscopical methods. Standing-wave microscopy has the potential for parallel super-resolution imaging as it simultaneously draws on SIM, PALM, and STED technologies Chen and Xi (2015).

Recently, methods were developed to expand biological specimens physically by synthesizing a swellable polymer network within the specimen. This process called expansion microscopy allows to separate labels spaced closer than the optical diffraction limit isotropically. Thus, super-resolution with diffraction-limited microscopes may be achieved (Chen et al., 2015, 2016; Engerer et al., 2016). However, it remains to be tested whether the swelling process is applicable to all organisms and tissues, amongst others those from plants, without disturbing the native structures. Additionally, the technique is not applicable for live cell imaging. Thus, it is not expected that expansion microscopy has the potential to replace optical nanoscopy. But both have the potential to be combined in some special applications.

Furthermore, with the development of genetically encoded markers for electron microscopy (Shu et al., 2011; Martell et al., 2012) correlative approaches with super-resolution techniques will become more powerful in near future. However, due to the harsher fixing conditions and the lack in high specificity electron microscopy will not have the potential to replace completely the super-resolution techniques as live cell imaging is not possible and multi-color labeling is a challenge.

## Author Contributions

VS conceived and designed the study, performed the experiments and wrote the manuscript.

## Conflict of Interest Statement

The author declares that the research was conducted in the absence of any commercial or financial relationships that could be construed as a potential conflict of interest. The reviewer FH and handling Editor declared their shared affiliation, and the handling Editor states that the process nevertheless met the standards of a fair and objective review.

## Abbreviations

CENH3: centromeric histone H3
FISH: fluorescence in situ hybridization
HILO: highly inclined and laminated optical sheet
PALM: Photoactivated Localization Microscopy
RNAPII: RNA polymerase II
SIM: structured illumination microscopy
STORM: stochastic optical reconstruction microscopy
STED: stimulated emission depletion
TIRF: total internal reflection fluorescence
